# An epigenetic biomarker for adult high-functioning autism spectrum disorder

**DOI:** 10.1038/s41598-019-50250-9

**Published:** 2019-09-20

**Authors:** Ryo Kimura, Masatoshi Nakata, Yasuko Funabiki, Shiho Suzuki, Tomonari Awaya, Toshiya Murai, Masatoshi Hagiwara

**Affiliations:** 1 Department of Anatomy and Developmental Biology, Graduate School of Medicine, Kyoto University, Kyoto, 606-8501 Japan; 2 Department of Cognitive and Behavioral Science, Graduate School of Human and Environmental Studies, Kyoto University, Kyoto, 606-8501 Japan; 3 Department of Psychiatry, Graduate School of Medicine, Kyoto University, Kyoto, 606-8507 Japan

**Keywords:** Diagnostic markers, Genetics research, Translational research

## Abstract

Increasing evidence suggests that epigenetic mechanisms play a role in the etiology of autism spectrum disorder (ASD). To date, several studies have attempted to identify epigenetic biomarkers for ASD. However, reliable markers remain to be established and most of these studies have focused on pediatric patients with ASD. In this study, we sought to find an epigenetic DNA methylation biomarker from peripheral blood for adult patients with high-functioning ASD. DNA methylation profiles were analyzed using the Illumina 450 K methylation array. To identify robust candidate markers, we employed two types of machine-learning algorithms for marker selection. We identified a potential marker (cg20793532) for which is the AUC value was 0.79. Notably, cg20793532 was annotated to the *PPP2R2C* gene, which was hypermethylated and down-regulated in blood from ASD patients compared to that in the controls. Although requiring careful interpretation, this pilot study seems to provide a potential blood biomarker for identifying individuals with high-functioning ASD.

## Introduction

Autism spectrum disorder (ASD) is a group of heterogeneous neurodevelopmental disorders with impaired social communication and behavioral problems^1^. Although the incidence of ASD is rapidly increasing globally, there are no blood-based diagnostic tools and no sufficient evidence regarding the efficacy of specific pharmacological treatments for the core symptoms of ASD^1–3^. Therefore, there is an urgent need for a reliable biomarker to assist in the diagnosis or drug discovery of ASD.

DNA methylation is one of the epigenetic factors involved in the regulation of gene expression in response to environmental influences without changing the DNA sequence^4^. Recently, accumulating evidence has indicated a role for DNA methylation in the etiology of ASD^5^. To date, four genome-wide DNA methylation studies of ASD using peripheral tissues have been reported^6–9^. Of these, three were small case-control studies using buccal epithelium (47 cases at age 7 and 48 controls at age 11)^8^, whole blood (50 monozygotic twin pairs at age 15)^7^, and lymphoblast cell line (7 discordant pairs of monozygotic autistic twins and 7 controls including unaffected siblings)^9^. The fourth study was a large case-control meta-analysis (796 cases and 858 controls; age 4–18)^6^, but subjects consisted of multiple ethnicities and were more than 80% male. To our knowledge, these attempts to identify the methylation markers of ASD from peripheral tissues have been challenging to reproduce mainly due to the heterogeneity of the disease phenotype and differences in clinical background. In addition, these studies were conducted in pediatric patients with ASD, and also insufficiently assessed the subjects’ intelligence quotient (IQ). Therefore, it still remains to be seen whether DNA methylation plays a role in adult ASD patients without intellectual disability.

In this preliminary study, we evaluated DNA methylation profiles between individuals with ASD and controls, especially focusing on high-functioning adults with ASD. To enhance robustness, we applied multiple experimental and computational approaches to age-, gender- and IQ-matched groups. We also examined the level of gene expression of the best DNA methylation marker and potential correlations between this marker and clinical severity of ASD. To our knowledge, this is the first study to explore an epigenetic biomarker in blood from high-functioning adult patients with ASD.

## Results

### Demographic and clinical profiles

In this study, we performed methylation arrays to compare high-functioning adults with ASD and age-, gender-, and IQ-matched controls in the discovery set. The Autism Diagnostic Observation Schedule (ADOS) and the Japanese version of the high-functioning Autism Spectrum Screening Questionnaire (ASSQ-R) were used to assess the presence and severity of ASD. Particularly, ASSQ-R is considered as a useful tool for assessing high-functioning ASD. As expected, both ADOS and ASSQ-R scores were significantly higher in patients with ASD than in controls (7.0 vs. 1.5, *P* = 1.04E-7 and 28.6 vs. 6.1, *P* < 2.2E-16, respectively). We used the replication set as an independent validation sample. Clinical parameters were similar in the discovery and replication set, but the replication set consisted of only male subjects. Demographic and clinical profiles of the participants are described in Supplementary Table S1.

### Genome-wide DNA methylation profiling

We assessed genome-wide DNA methylation in the discovery set and found significantly altered methylation profiles spread across the genome between patients with ASD and controls (Supplementary Fig. S1). To assess for the effect of the blood cell composition, we applied the reference-based Houseman algorithm to estimate the proportions of six blood cell subtypes (B cells, CD8 T cells, CD4 T cells, natural killer cells, monocytes, and granulocytes)^10^. No significant differences were observed in blood cell composition between patients with ASD and controls (Supplementary Fig. S2). For further analysis, we selected as Differentially methylated positions (DMPs) a total of 50 CpG probes with FDR of 0.1 or less and absolute beta differences of 0.1 or greater (Supplementary Table S2).

### DNA methylation-based classification

**Figure 1:**
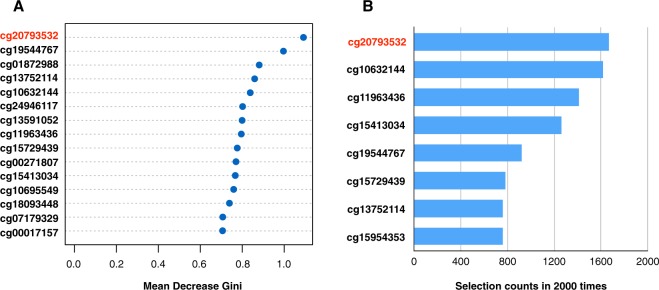
The classification performance based on methylation profiling was performed with two machine-learning algorithms. (**A**) Variable importance ranking with the Mean Decrease Gini index calculated by the random forest (RF) algorithm. The 15 highest importance values are shown. (**B**) The Greedy Algorithm was applied to 2000 selections of random 80% subsets of samples. The eight most frequently chosen markers are shown with their selection rates.

To identify robust candidate markers, two different types of machine-learning techniques were applied to classify patients with ASD and controls. The random forest and the Coarse Approximation Linear Function (CALF) algorithms were applied to rank the importance of 50 DMPs from methylation data. The random forest analysis provided the mean decrease Gini (MDG), which was used as a measure of the importance of a variable for distinguishing patients with ASD from controls. The MDG is the sum of all the these decreases due to a given variable, normalized by the number of trees in the forest^11,12^. The random forest analysis results are summarized in ranked order in Fig. 1A. The CALF algorithm was developed from all subjects using the sum of the first 10 DMPs chosen (AUC = 0.99). Testing this algorithm applied to randomized data resulted in an empirical *P*-value of 5.0E-4. We then applied CALF to 2000 random selections of 80% subsets of ASD patients and 80% subsets of controls. The 10 DMPs were selected in at least 750 of the 2000 trials, and then 8 of these were also among the 10 DMPs in the initial classifier developed from all subjects (Fig. 1B). Based on these two approaches, we selected cg20793532 as the most reliable CpG among candidate markers (Fig. 1). This CpG site was annotated to the gene body of the *PPP2R2C* gene. Methylation levels at cg20793532 were significantly different between patients with ASD and controls (FDR = 5.66E-3, mean beta differences = 0.10) (Supplementary Table S2).

### Validation by pyrosequencing

**Figure 2:**
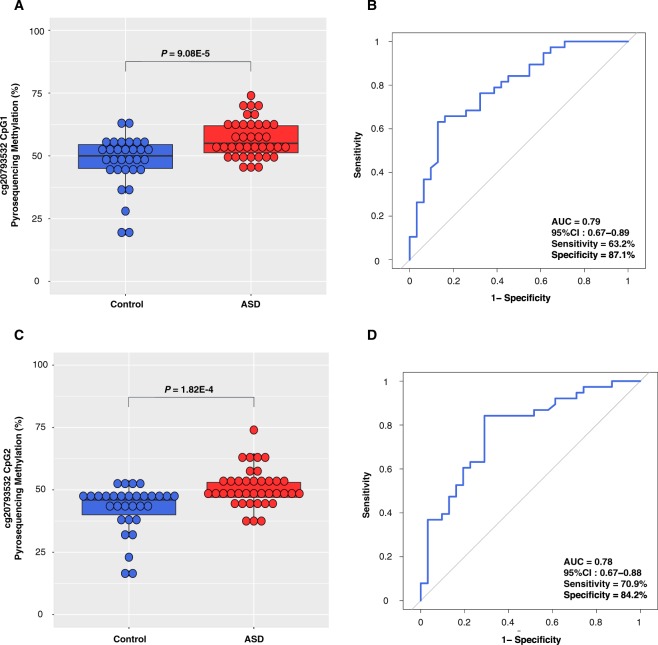
Pyrosequencing validation of cg20793532. (**A**) Comparison of methylation levels of cg20793532 CpG1 between controls and ASD patients in the discovery set. Boxplots represent pyrosequencing-based methylation levels. The line within the box represents the median. Student’s *t*-test showed significant differences (*P* = 9.08E-5). (**B**) The receiver operating characteristic (ROC) curve analysis for evaluating the diagnostic performance of the methylation levels of cg20793532 CpG1. AUC with 95% confidence intervals (95% CI) was calculated (AUC = 0.79, 95% CI: 0.67–0.89). (**C**) Comparison of methylation levels of cg20793532 CpG2 between controls and ASD patients in the discovery set. Student’s *t*-test showed significant differences (*P* = 1.82E-4). (**D**) The ROC curve analysis of the methylation levels of cg20793532 CpG2. AUC with 95% confidence intervals (95% CI) was calculated (AUC = 0.78, 95% CI: 0.67–0.88).

To validate the top CpG site (cg20793532) identified by methylation array analysis, we performed pyrosequencing. Two CpGs (CpG1 and CpG2) were covered in this pyrosequencing. We found that cg20793532 CpG1 was significantly hypermethylated in patients with ASD (mean 57.0%; median 55.0%) compared to that in the controls (mean 47.9%; median 50.0%) in the discovery set (*P* = 9.08E-5) (Fig. 2A). The receiver operating characteristic (ROC) curve analysis for cg20793532 CpG1 revealed an area under the curve (AUC) of 0.79 with a sensitivity of 63.2% and a specificity of 87.1% (Fig. 2B). We also found that cg20793532 CpG2 was significantly hypermethylated in patients with ASD (mean 50.6%; median 49.0%) compared to that in the controls (mean 42.5%; median 46.0%) (*P* = 1.82E-4) (Fig. 2C). The ROC curve analysis for cg20793532 CpG2 revealed an AUC of 0.78, with a sensitivity of 70.9% and a specificity of 84.2% (Fig. 2D). Next, we sought to confirm these results using an independent sample set. We found that both cg20793532 CpG1 (mean 55.6%; median 54.0%) and CpG2 (mean 50.0%; median 50.0%) were significantly hypermethylated in patients with ASD compared to that in the controls (mean 50.6% and 46.1%, respectively; median 50.5% and 48.0%, respectively) in the replication set (P = 0.02 and 0.04, respectively) (Supplementary Fig. S3A,C). The ROC curve analysis for both cg20793532 CpG1 and CpG2 in the replication set showed an AUC value of 0.78, with sensitivities of 81.8 and 80.0%, respectively and specificities of 70.0 and 72.7%, respectively (Supplementary Fig. S3B,D). Together, these validation results indicated the robustness of our approach and its potential as a biomarker.

### Correlation with clinical severity of ASD

Next, we investigated the correlation between the methylation levels of the top CpG site (cg20793532), as determined by pyrosequencing, and the clinical features in the discovery set. We found no statistically significant correlation between the methylation levels of cg20793532 (both CpG1 and CpG2) and the total score of ASSQ-R, total score of ADOS, and IQ (Supplementary Fig. S4). These results suggested that our identified epigenetic marker seems to be independent of clinical severity.

### Differential gene expression of *PPP2R2C*

**Figure 3:**
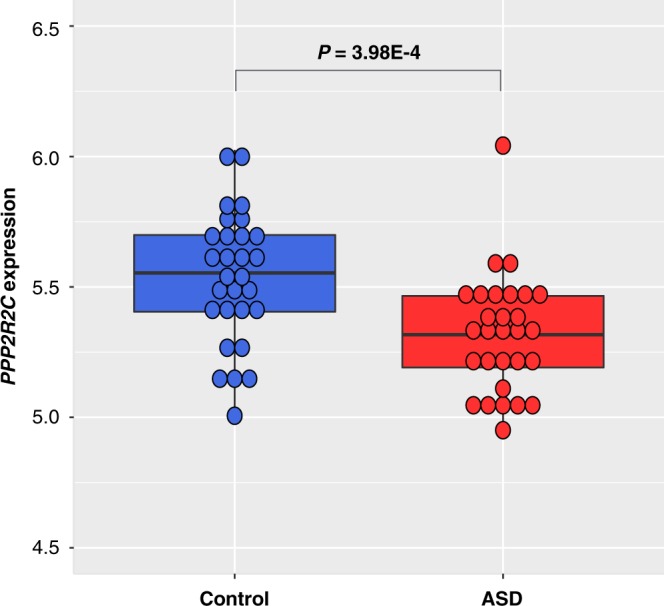
Differential gene expression of *PPP2R2C*. *PPP2R2C* gene was significantly down-regulated in blood from ASD patients (n = 29) compared to that in the controls (n = 29). Microarray expression data was obtained from Kimura *et al*.^33^ Student’s *t*-test showed significant differences (*P* = 3.98E-4).

Finally, we investigated the regulation of gene expression by methylation of *PPP2R2C* using our previous microarray expression profiling data. Notably, we found that the *PPP2R2C* gene was significantly down-regulated in blood from ASD patients compared to that in the controls (*P* = 3.98E-4) (Fig. 3). To examine the expression levels in other tissue types, we measured the *PPP2R2C* gene expression in normal human tissues. Although we detected some *PPP2R2C* expression in skin and kidney, expression was considerably enriched in the brain (Supplementary Fig. S5).

## Discussion

To our knowledge, this is the first study to explore an epigenetic biomarker in blood from high-functioning adults with ASD. Through multiple approaches, we identified a potential marker (cg20793532) for which is the AUC value was 0.79. Notably, cg20793532 was annotated to the *PPP2R2C* gene, which was hypermethylated and down-regulated in blood from ASD patients compared to that in the controls. Although preliminary due to sample size, our findings indicated that DNA methylation in the peripheral blood could potentially be applied to develop a tool for diagnosing high-functioning ASD.

The machine-learning approach shows greater robustness in exploring potential biomarkers for disease diagnosis and prognosis than conventional methods in case of handling large complex data sets^13^. Particularly, decision tree algorithms have been widely exploited for classifying biological samples based on various biomarkers^14^. In this study, two different types of decision tree algorithms, Random Forest and CALF, were employed. Random Forest is currently a popular and efficient decision tree algorithm for classification^15^. CALF is also a type of decision tree approach based on a greedy forward-stepwise regression algorithm, and has been used to successfully identify biomarkers for psychosis^16^. However, as machine-learning techniques are a rapidly growing area in many research fields, it should be noted that more excellent methods may emerge in the near future.

Recent report using peripheral blood from a large ASD cohort aged under 18 showed that ASD was not associated with DNA methylation at a genome-wide significance based on a Bonferroni corrected *P*-value^6^. It should be noted that this result was obtained from individuals of different ethnic backgrounds. It is known that potential confounding factors, such as ethnicity, cigarette smoking, and medication use, influence on epigenetic changes^17^. Therefore, we considered reducing confounding factors including age, gender, IQ, smoking and medication status between patients with ASD and controls in this study. Although sample size was small, our results showed that this approach is capable of capturing a potential epigenetic marker.

PPP2R2C encodes a gamma isoform of the subunit B55 subfamily, which is a regulatory subunit of protein phosphatase type 2 A (PP2A)^18^. In accordance with our results, which demonstrate that the expression of the *PPP2R2C* gene was enriched in the human brain, a previous study on the mouse brain has suggested that the expression of the PPP2R2C gene may play a role in synaptic plasticity^19^. In addition, a preliminary report indicates that the *PPP2R2C* gene could increase vulnerability for attention-deficit/hyperactivity disorder (ADHD)^20^. Moreover, dysregulation of other B regulatory subunits of PP2A, which modulate tau phosphorylation, have been implicated in neurodegenerative disorders such as Alzheimer’s disease^21,22^. Although the exact function has not yet been elucidated, these findings support a key role for PPP2R2C in cognitive processes and behavior.

This study has some limitations that should be addressed. First, this study had a relatively small sample size, and the replication sample consisted of only male subjects. Second, participants in this study included only Japanese individuals from a single university hospital. Third, the AUC value demonstrates that our features are not perfect and achieved moderate accuracy. Fourth, we did not correct for several covariates in the initial screening with methylation array because we were concerned about the possibility of missing candidate markers due to overly rigorous correction. Adjustment for covariates including cell-type composition, age, and sex is considered critical for interpretation and understanding of genome-wide DNA methylation studies^17^. To address this issue, we compared the original values to those corrected for these covariates. Most of the top DMPs in the original data displayed similar trends and remained significant even after correction for covariates (Supplementary Table S2). However, it should be noted that quantile-quantile plots (QQ-plots) exhibited a tendency toward mild inflation (Supplementary Fig. S6). Finally, we used the CALF algorithm for marker selection. To our knowledge, this approach has previously only been applied to miRNA expression data and has not been reported for methylation studies^16,23^. Therefore, this algorithm may require more optimized algorithms and parameter settings for methylation analysis. Further studies from multiple different institutions with a larger sample size and with appropriate methods to control for covariates would be needed to confirm our findings.

In summary, we explored an epigenetic DNA methylation biomarker for adults with high-functioning ASD from peripheral blood. Our identified CpG site (cg20793532) within the *PPP2R2C* gene has potential as an epigenetic biomarker, and was significantly hypermethylated in patients with ASD compared to controls. These preliminary findings may provide evidence for developing diagnostic biomarkers for adult high-functioning ASD.

## Methods

### Participants

We recruited patients with ASD from the Department of Psychiatry at Kyoto University Hospital and healthy individuals from among community volunteers. Participants were divided into a discovery and a replication set. The diagnosis of ASD was based on a psychiatric interview according to the fifth edition of the Diagnostic and Statistical Manual of Mental Disorders (DSM-5) criteria, and was confirmed with the Autism Diagnostic Observation Schedule (ADOS) instruments and/or the Japanese version of the high-functioning Autism Spectrum Screening Questionnaire (ASSQ-R)^24,25^. Participants IQs were assessed using the third edition of Wechsler Adult Intelligence Scale^26^. Exclusion criteria for all participants included IQ < 80; any additional psychiatric or neurologic disorder (including history of seizures) and other medical disorders; and cigarette smoking or psychotropic medication use for at least three months before the collection of blood samples. This study was approved by the ethics committee of the Graduate School of Medicine, Kyoto University, and informed consent was obtained from all participants in accordance with the Declaration of Helsinki. All methods were performed in accordance with the relevant guidelines and regulations.

### DNA methylation analysis

Whole blood samples were collected in EDTA tubes and genomic DNA was extracted with a QIAamp DNA Blood Midi Kit (Qiagen, Tokyo). The quality of DNA samples was assessed using a NanoDrop 2000 spectrophotometer (Thermo Fisher Scientific, Yokohama, Japan) and Agilent 2200 TapeStation System with Genomic DNA Screen Tape (Agilent Technologies, Tokyo). For each sample, 500 ng of DNA was bisulfite converted using an EZ DNA Methylation kit (Zymo Research, Irvine, CA). DNA methylation levels were assessed using the Illumina Infinium HumanMethylation450 BeadChip array, which includes over 485,000 CpG sites across the entire genome (Illumina, Tokyo). Hybridization was performed according to the manufacturer’s protocol. The methylation arrays were scanned using an Illumina iScan (Illumina) and the signal intensities were quantified using GenomeStudio software (Illumina). Data quality control and analysis were performed using the ChAMP methylation analysis package (version 2.6.4) in R^27^. Briefly, probe filtering was applied to remove probes that failed to hybridize with a detection *P*-value above 0.01 in one or more samples and had a bead count of less than 3 in 5% of samples. Subsequently, probes on sex chromosomes, probes containing single nucleotide polymorphisms and non-specific binding probes were also filtered out. To correct the beta value distributions of the two types of probes, the Beta Mixture Quantile Dilation (BMIQ) method was used^28^. After quality control, 410,559 CpG probes were retained for further analysis. Singular value decomposition (SVD) was used to identify batch effects and the ComBat method was used to correct for effects related to sex and array. The cell-type composition was estimated by Houseman’s algorithm in R^10^. Differentially methylated positions (DMPs) were identified using the Limma R package^29^.

### Pyrosequencing analysis

Bisulfite conversion was performed on 250 ng of genomic DNA with the EpiTect Fast DNA Bisulfite Kit (Qiagen) and then 20 ng of the converted DNA was amplified by PCR using the PyroMark PCR kit (Qiagen) and the designed primers (Supplementary Table S3). PCR and sequencing primers were designed using PyroMark Assay Design Software 2.0 (Qiagen). PCR conditions were as follows: 95 °C for 15 minutes; 94 °C for 30 seconds, 56 °C for 30 seconds, and 72 °C for 30 seconds for 45 cycles; 72 °C for 10 minutes; and 4 °C hold. Pyrosequencing was performed on the PyroMark Q96 ID system with the PyroMark Q96 Vacuum Workstation and PyroMark Gold Q96 Reagents (Qiagen) according to the manufacturer’s instructions. Pyrosequencing data were analyzed using the PyroMark Q96 software, version 2.5.8 (Qiagen).

### Machine-learning algorithms

Two machine-learning algorithms were implemented in this study to perform a binary classification (ASD vs. controls) using DNA methylation data. We used the randomForest R package (version 4.6.12)^30^ and the Coarse Approximation Linear Function (CALF) R package (version 0.1.3)^16^ for feature selection and classification analysis. The Random forest algorithm is a supervised classification method based on an ensemble of decision trees that estimate the importance of variables to the classification of subjects^30^. Variable importance is described by the mean decrease in Gini Index^31^. The number of trees was set to 500, and the size of the predictor subset (mtry) was optimized using repeated 10-fold cross-validation. The CALF is a type of forward selection linear regression greedy algorithm^16,23^. This algorithm first selected the best predictor (differentially methylated CpG site) to discriminate between patients with ASD and controls, based on a Pearson correlation, then CpG probes were added to the model until no improvement in the metric was possible. Following previous studies^16,23^, the weights of selected DMPs were either +1 or −1, and then the greedy algorithm was applied 2000 times to randomized data to validate the statistical significance of selected DMPs. The accuracy of predictions was evaluated by the area under the curve (AUC) of the receiver operating characteristic (ROC) after adjustment for age and gender in generalized linear models (GLM) using the pROC R package (version 1.8)^32^. The 95% confidence intervals (CI) were calculated using a bootstrapping method with 2000 replications.

### Gene expression analysis

Gene expression data were obtained from our previous microarray study of blood samples from ASD patients and age-gender-race matched controls (GSE89594)^33^. Samples from gene expression data were seen to partially overlap with those from methylation analysis (overlap ratio; ASD 62.1%, Control 86.2%).

### Statistical analysis

Data were analyzed using the Student’s *t*-test and the correlation between two variables was analyzed using Pearson’s correlation coefficient. Differences with a *P*-value of less than 0.05 were considered statistically significant. Multiple testing correction for array data was performed by the Benjamini-Hochberg (BH) procedure for false discovery rate (FDR) adjustment^34^.

### Ethics approval and consent to participate

This study was approved by the ethics committee of Kyoto University Graduate School and Faculty of Medicine, and informed consent was obtained from all participants in accordance with the Declaration of Helsinki.

## Supplementary information

Supplementary information

## Data Availability

The DNA methylation data set was deposited in the NCBI Gene Expression Omnibus (GEO) under Accession Number GSE109905. Computer code used in this study is available on request.
