# Supplementary material for: An epigenetic biomarker for adult high-functioning autism spectrum disorder
Source: Sci Rep. 2019 Sep 20;9:13662. doi: 10.1038/s41598-019-50250-9 (PMC6754433; doi:10.1038/s41598-019-50250-9)
Supplement: Supplementary file 1 — Supplementary information [file 41598_2019_50250_MOESM1_ESM.docx]

**Supplementary information**

**An epigenetic biomarker for adult high-functioning autism spectrum disorder**

Ryo Kimura, Masatoshi Nakata, Yasuko Funabiki, Shiho Suzuki, Tomonari Awaya, Toshiya Murai, Masatoshi Hagiwara

**Methods**

Methylation analysis with correction for covariates

After quality control, 410,559 CpG probes were used to assess and correct batch effects (sex and array). Blood cell mixture composition was estimated by using the Houseman algorithm. To control for the effects of age, sex and blood cell type composition, the R package “limma” was used to compute a linear regression adjusted for these factors.

Human samples

The following commercially available total RNA samples from the Human Adult Normal Tissue 5 Donor Pool were used: brain (cat. R1234035-P), frontal lobe (cat. R1234051-P), occipital lobe (cat. R1234062-P), parietal lobe (cat. R1234066-P), temporal lobe (cat. R1234078-P), skin (cat. R1234218-P), heart (cat. R1234122-P), kidney (cat. R1234142-P), spleen (cat. R1234246-P), lung (cat. R1234152-P) and liver (cat. R1234149-P). The samples were obtained from BioChain (Newark, CA, USA).

Real-time qRT-PCR

Reverse transcription of total RNA was performed using a High Capacity cDNA Reverse Transcription Kit (Thermo Fisher Scientific, Yokohama, Japan). Gene expression was analyzed using TaqMan Master Mix and TaqMan Assay Reagents (Thermo Fisher Scientific). Real-time PCR reactions were run in duplicate using an ABI 7900HT Fast Real-Time PCR System, and data were processed using the Sequence Detection System (SDS) software version 2.4 (Thermo Fisher Scientific), for relative quantification. The relative expression was calculated and normalized relative to GAPDH using the ddCt method. All primers used in the study are listed in Supplementary Table 3.


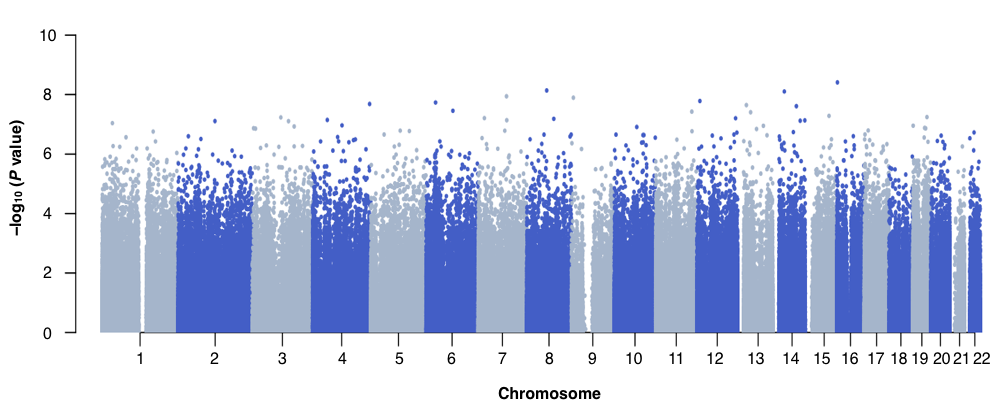


**Supplementary Figure S1**

Genome-wide DNA methylation profiling

Manhattan plot of the genome-wide DNA methylation analysis in whole blood from patients with autism spectrum disorder (ASD) and controls. The figure shows the −log10 *P* values of the association of CpG sites with ASD according to the chromosome number.


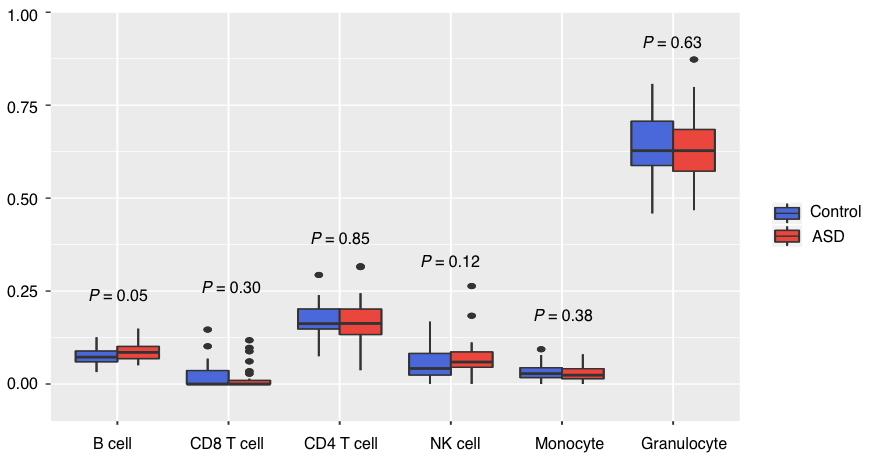


**Supplementary Figure S2**

Blood cell-type composition.

The proportions of six blood cell subtypes [B cells, CD8 T cells, CD4 T cells, natural killer (NK) cells, monocytes, and granulocytes] were estimated using the results of the DNA methylation array. Student's *t*-test was used for statistical analysis of the differences between controls and patients with autism spectrum disorder (ASD).


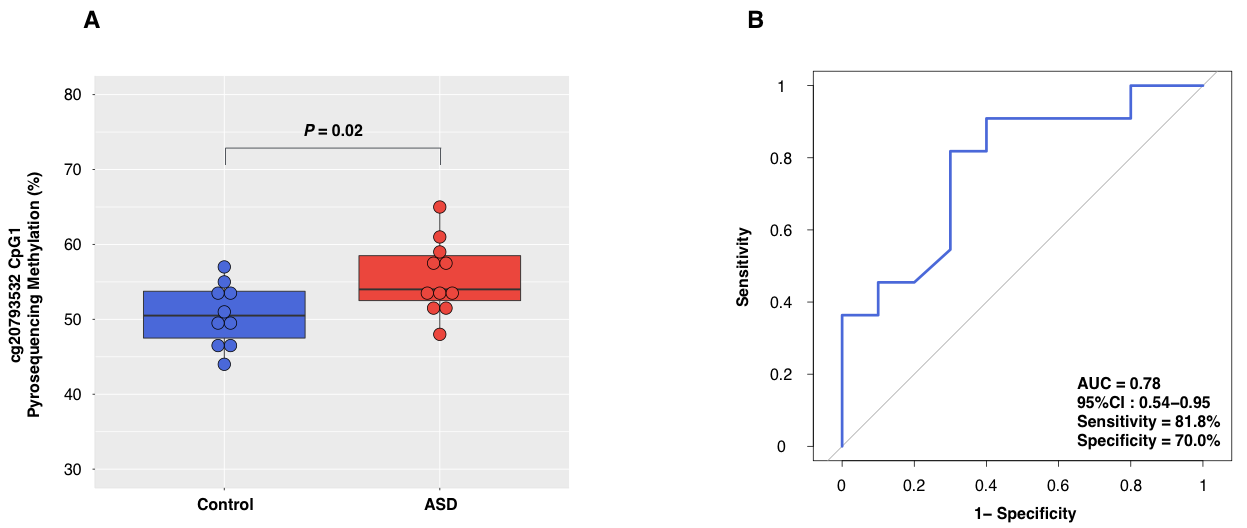


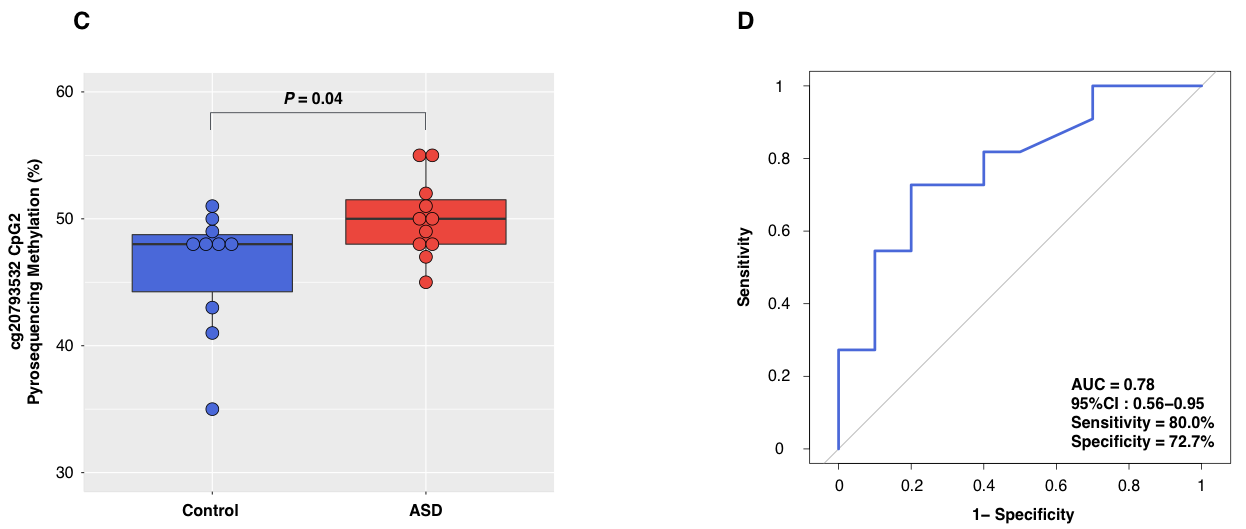


**Supplementary Figure S3**

Pyrosequencing validation of cg20793532 using an independent sample set

(A) Comparison of the methylation levels of cg20793532 CpG1 between controls and ASD patients in the replication set. Boxplots represent pyrosequencing-based methylation levels. The line within the box represents the median value. Student's t-test showed significant differences (*P* = 0.02).

(B) The receiver operating characteristic (ROC) curve analysis for evaluating the diagnostic performance of the methylation levels of cg20793532 CpG1. AUC with 95% confidence intervals (95% CI) was calculated (AUC = 0.78, 95% CI: 0.54-0.95).

(C) Comparison of the methylation levels of cg20793532 CpG2 between controls and ASD patients in the replication set. Student's t-test showed significant differences (*P* = 0.04).

(D) The ROC curve analysis of the methylation levels of cg20793532 CpG2. AUC with 95% confidence intervals (95% CI) was calculated (AUC = 0.78, 95% CI: 0.56-0.95).


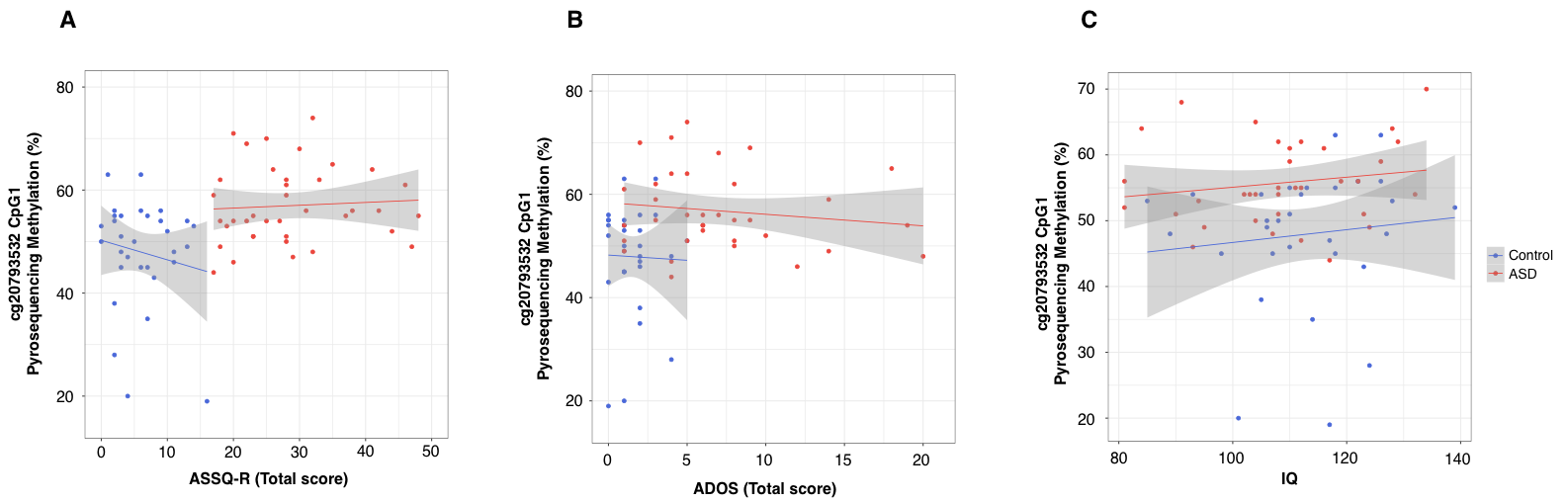


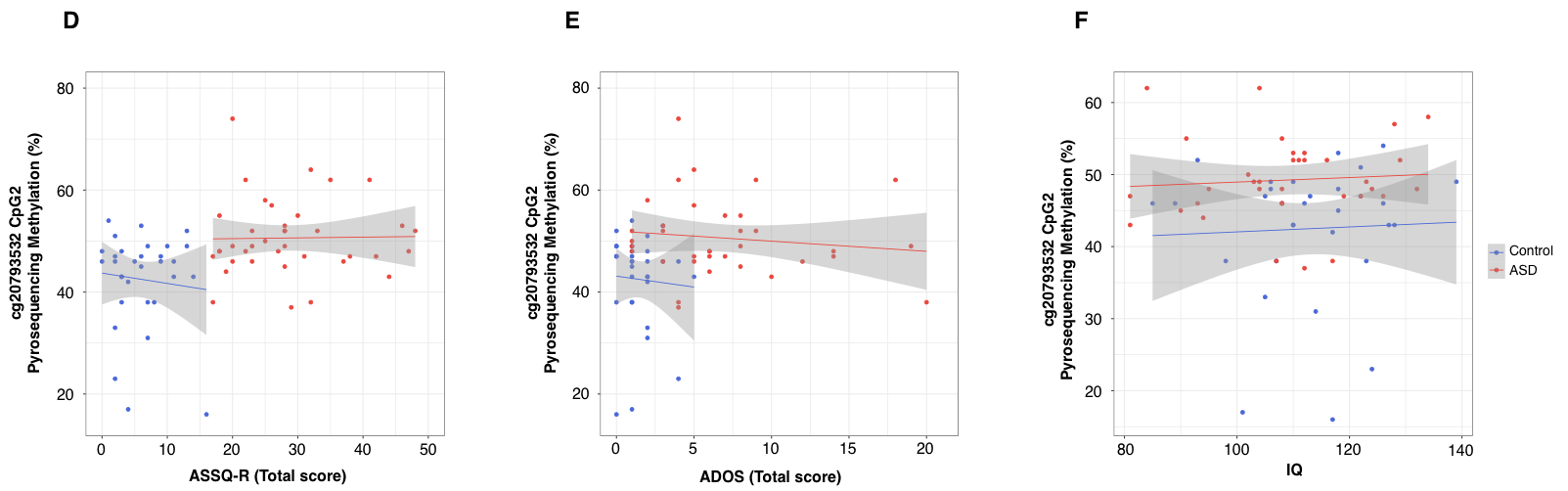


**Supplementary Figure S4**

The clinical severity scores were not significantly correlated with methylation levels of cg20793532.

(A-C) Correlation between the clinical features and methylation status of cg20793532 CpG1.

(D-F) Correlation between the clinical features and methylation status of cg20793532 CpG2.

(A, D) The total score of high-functioning Autism Spectrum Screening Questionnaire (ASSQ-R)

(B, E) The total score of Autism Diagnostic Observation Schedule (ADOS)

(C, F) The total score of intelligence quotient (IQ)

**
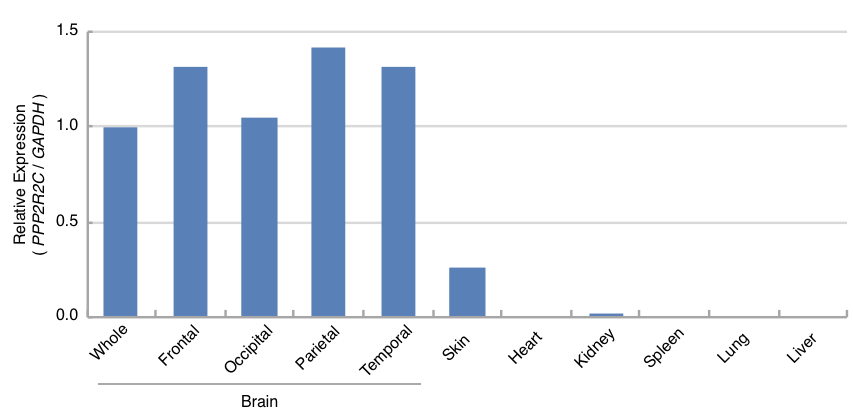
**

**Supplementary Figure S5**

Real-time qRT-PCR analysis of the organ distribution of *PPP2R2C* gene expression.

All reactions were run in duplicate and GAPDH served as an internal control.


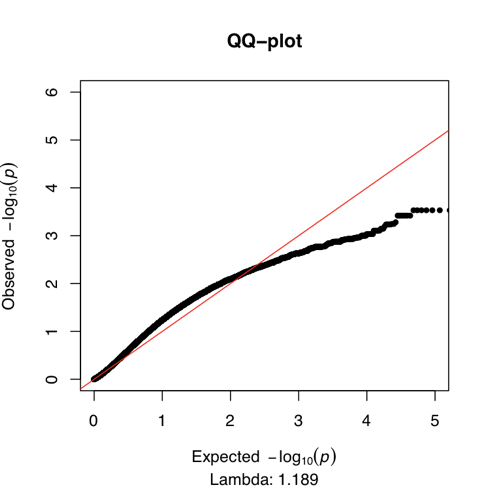


**Supplementary Figure S6**

A quantile-quantile (QQ) plot representing the expected and observed p-values.

QQ plot displayed after adjustment for covariates including cell-type composition, age and sex.

**Supplementary Table S1.** Demographic and clinical profiles of participants.

|  | Discovery set | | |  | Replication set | | |
| --- | --- | --- | --- | --- | --- | --- | --- |
|  | ASD | Control | *P*-value |  | ASD | Control | *P*-value |
| Individuals, n | 38 | 31 |  |  | 11 | 10 |  |
| Age, mean years | 28.6 (6.5) | 26.7 (6.7) | 0.24 |  | 34.5 (10.3) | 31.2 (10.0) | 0.47 |
| Gender, male (%) | 23 (60.5) | 16 (51.6) | 0.46 |  | 11 (100) | 10 (100) |  |
| Race (% Japanese) | 100 | 100 |  |  | 100 | 100 |  |
| Smoker, n | 0 | 0 |  |  | 0 | 0 |  |
| WAIS-Ⅲ |  |  |  |  |  |  |  |
| Total IQ score | 107.8 (14.7) | 112.8 (12.1) | 0.05 |  | 108.2 (14.7) | 121.1 (10.9) | 0.04 |
| ADOS |  |  |  |  |  |  |  |
| Total score | 7.0 (5.0) | 1.5 (1.3) | <0.05 |  | 11.2 (4.7) | 1.9 (1.9) | <0.05 |
| ASSQ-R |  |  |  |  |  |  |  |
| Total score | 28.6 (9.0) | 6.1 (4.4) | <0.05 |  | − | − | − |

WAIS-III = third edition of Wechsler Adult Intelligence Scale; IQ = intelligence quotient; ADOS = Autism Diagnostic Observation Schedule; ASSQ-R = Autism Spectrum Screening Questionnaire.

**Supplementary Table S2.** Lists of differentially methylated positions (DMPs) between ASD and controls [FDR-adjusted *P*-value < 0.1, beta difference (Δβ) > 0.1].

TSS1500, 200–1500 bases upstream from the transcriptional start site (TSS); TSS200, 0–200 bases up- stream from the TSS; 1st Exon; 5′UTR, 5′ untranslated region; body, gene body; 3′UTR, 3′ untranslated region; IGR, intergenic region.

|  |  |  |  | Original analysis | | | Corrected analysis | | |
| --- | --- | --- | --- | --- | --- | --- | --- | --- | --- |
| Probe ID | Gene symbol | Chr | Feature | Δβ | *P-*value | adj *P-*value | Δβ | *P-*value | adj *P-*value |
| cg15413034 |  | 19 | IGR | 0.103 | 3.58E-06 | 5.11E-03 | 0.070 | 6.13E-05 | 7.28E-03 |
| cg01872988 | *DKFZp686A1627* | 13 | TSS1500 | 0.329 | 4.74E-06 | 5.58E-03 | 0.249 | 1.47E-04 | 1.02E-02 |
| **cg20793532** | ***PPP2R2C*** | 4 | Body | 0.104 | 5.17E-06 | 5.66E-03 | 0.080 | 2.09E-05 | 4.83E-03 |
| cg10632144 | *EBPL* | 13 | Body | -0.101 | 8.91E-06 | 6.45E-03 | -0.091 | 9.00E-06 | 3.67E-03 |
| cg19544767 |  | 13 | IGR | 0.119 | 2.38E-05 | 8.78E-03 | 0.096 | 2.12E-05 | 4.85E-03 |
| cg24946117 |  | 11 | IGR | 0.127 | 5.88E-05 | 1.13E-02 | 0.093 | 4.84E-04 | 1.71E-02 |
| cg11963436 | *TNKS2* | 10 | Body | -0.106 | 7.04E-05 | 1.20E-02 | -0.087 | 1.60E-04 | 1.06E-02 |
| cg16034541 | *MUC4* | 3 | Body | 0.149 | 1.95E-04 | 1.73E-02 | 0.113 | 6.57E-04 | 1.97E-02 |
| cg03874092 | *BCL11A* | 2 | 1stExon | 0.100 | 3.96E-04 | 2.29E-02 | 0.071 | 1.99E-03 | 3.35E-02 |
| cg00271807 | *LOC440905* | 2 | Body | 0.111 | 5.77E-04 | 2.66E-02 | 0.069 | 4.98E-03 | 5.37E-02 |
| cg16720578 |  | 14 | IGR | 0.129 | 6.67E-04 | 2.82E-02 | 0.114 | 3.28E-04 | 1.45E-02 |
| cg04350215 | *ABCA4* | 1 | Body | 0.140 | 8.07E-04 | 3.05E-02 | 0.111 | 4.10E-03 | 4.84E-02 |
| cg04758026 |  | 2 | IGR | 0.124 | 8.69E-04 | 3.14E-02 | 0.095 | 3.59E-03 | 4.52E-02 |
| cg12568536 |  | 11 | IGR | 0.242 | 1.31E-03 | 3.76E-02 | 0.202 | 2.07E-03 | 3.42E-02 |
| cg00009523 | *SNTG2* | 2 | Body | 0.226 | 1.35E-03 | 3.80E-02 | 0.217 | 7.98E-04 | 2.16E-02 |
| cg07620853 | *GAD1* | 2 | Body | 0.164 | 1.36E-03 | 3.81E-02 | 0.133 | 3.44E-03 | 4.43E-02 |
| cg00876757 |  | 16 | IGR | 0.113 | 2.06E-03 | 4.57E-02 | 0.049 | 5.91E-02 | 2.11E-01 |
| cg14464852 | *TCERG1L* | 10 | Body | 0.169 | 2.13E-03 | 4.63E-02 | 0.146 | 2.53E-03 | 3.78E-02 |
| cg19498042 | *TMPRSS7* | 3 | TSS1500 | 0.108 | 2.13E-03 | 4.64E-02 | 0.069 | 1.28E-02 | 8.91E-02 |
| cg04506342 | *BAZ2B* | 2 | 5'UTR | 0.161 | 2.76E-03 | 5.17E-02 | 0.145 | 2.13E-03 | 3.47E-02 |
| cg11706129 |  | 1 | IGR | 0.102 | 2.86E-03 | 5.26E-02 | 0.101 | 7.53E-04 | 2.10E-02 |
| cg05122437 | *MTUS2* | 13 | Body | 0.100 | 3.22E-03 | 5.56E-02 | 0.083 | 4.46E-03 | 5.07E-02 |
| cg19235645 | *FAM101A* | 12 | 5'UTR | 0.218 | 3.49E-03 | 5.77E-02 | 0.165 | 9.74E-03 | 7.66E-02 |
| cg06711418 | *MT2A* | 16 | 3'UTR | 0.157 | 3.52E-03 | 5.80E-02 | 0.140 | 2.17E-03 | 3.50E-02 |
| cg18918831 | *MUC4* | 3 | Body | 0.115 | 3.66E-03 | 5.89E-02 | 0.090 | 4.05E-03 | 4.81E-02 |
| cg07474670 | *NCOR2* | 12 | Body | -0.204 | 3.81E-03 | 6.01E-02 | -0.176 | 7.43E-03 | 6.62E-02 |
| cg17395555 |  | 5 | IGR | 0.124 | 3.83E-03 | 6.03E-02 | 0.100 | 5.77E-03 | 5.79E-02 |
| cg15717617 | *PLEKHA2* | 8 | Body | 0.124 | 3.93E-03 | 6.09E-02 | 0.092 | 7.78E-03 | 6.78E-02 |
| cg15729439 |  | 11 | IGR | 0.105 | 4.22E-03 | 6.29E-02 | 0.095 | 1.49E-03 | 2.91E-02 |
| cg18093448 | *WWC2* | 4 | Body | -0.150 | 4.23E-03 | 6.30E-02 | -0.148 | 8.01E-04 | 2.16E-02 |
| cg08176410 | *IPO5* | 13 | TSS200 | 0.162 | 4.68E-03 | 6.59E-02 | 0.129 | 1.25E-02 | 8.78E-02 |
| cg26294955 |  | 5 | IGR | 0.105 | 5.01E-03 | 6.80E-02 | 0.091 | 4.53E-03 | 5.11E-02 |
| cg10288111 | *IFRD1* | 7 | TSS1500 | 0.107 | 5.01E-03 | 6.80E-02 | 0.096 | 2.16E-03 | 3.49E-02 |
| cg13591052 |  | 5 | IGR | 0.112 | 5.15E-03 | 6.88E-02 | 0.092 | 4.30E-03 | 4.97E-02 |
| cg20364183 | *LSAMP* | 3 | Body | 0.110 | 5.68E-03 | 7.20E-02 | 0.059 | 8.07E-02 | 2.53E-01 |
| cg05834845 | *MUC4* | 3 | Body | 0.113 | 5.76E-03 | 7.25E-02 | 0.096 | 3.99E-03 | 4.77E-02 |
| cg13752114 | *MUC4* | 3 | Body | 0.106 | 6.24E-03 | 7.52E-02 | 0.081 | 5.54E-03 | 5.67E-02 |
| cg15954353 | *LOC728392* | 17 | 3'UTR | -0.129 | 6.45E-03 | 7.63E-02 | -0.117 | 4.09E-03 | 4.83E-02 |
| cg16462528 | *LECT1* | 13 | Body | 0.120 | 6.83E-03 | 7.84E-02 | 0.115 | 1.74E-03 | 3.14E-02 |
| cg07179329 | *CDH13* | 16 | Body | 0.121 | 7.26E-03 | 8.06E-02 | 0.108 | 4.70E-03 | 5.22E-02 |
| cg09829645 | *DYSF* | 2 | Body | 0.166 | 7.79E-03 | 8.34E-02 | 0.127 | 1.42E-02 | 9.45E-02 |
| cg26772116 | *MTIF3* | 13 | 5'UTR | 0.115 | 9.13E-03 | 9.01E-02 | 0.085 | 2.93E-02 | 1.41E-01 |
| cg10695549 | *PSD3* | 8 | Body | 0.179 | 9.25E-03 | 9.06E-02 | 0.143 | 1.73E-02 | 1.05E-01 |
| cg26398228 |  | 4 | IGR | 0.166 | 9.33E-03 | 9.10E-02 | 0.180 | 1.83E-03 | 3.22E-02 |
| cg25243082 |  | 4 | IGR | -0.121 | 9.49E-03 | 9.17E-02 | -0.094 | 1.45E-02 | 9.55E-02 |
| cg22543924 |  | 12 | IGR | 0.208 | 9.55E-03 | 9.20E-02 | 0.187 | 1.04E-02 | 7.93E-02 |
| cg23649088 | *C2orf69* | 2 | TSS1500 | -0.124 | 1.05E-02 | 9.62E-02 | -0.133 | 1.27E-03 | 2.69E-02 |
| cg04131969 | *MYADML* | 2 | Body | 0.199 | 1.06E-02 | 9.68E-02 | 0.158 | 3.07E-02 | 1.45E-01 |
| cg26512469 | *MCF2L* | 13 | Body | -0.155 | 1.08E-02 | 9.77E-02 | -0.126 | 1.33E-02 | 9.10E-02 |
| cg00017157 |  | 1 | IGR | 0.213 | 1.09E-02 | 9.80E-02 | 0.226 | 3.03E-03 | 4.14E-02 |

**Supplementary Table S3.**

A) Pyrosequencing primer sequences for PPP2R2C

| Forward primer | GTTGGGGGTTTATGAAGGGT |  |
| --- | --- | --- |
| Reverse primer | ATCCTCAAAATAAACAATTACTTCTACTT | Biotin 5’ |
| Sequencing Primer | TTTTAATTTTAAGAAATTTTAGTG |  |

B) List of Taqman probes for real-time RT-PCR analysis.

| Gene Symbol | Assay ID | Gene Name |
| --- | --- | --- |
| GAPDH | Hs02758991_g1 | glyceraldehyde-3-phosphate dehydrogenase |
| PPP2R2C | Hs00739033_m1 | protein phosphatase 2 regulatory subunit Bgamma |
